# 2230. Consumption of Quinolones, Macrolides, and Beta-Lactam Antibiotics at a Tertiary Care Veterans Affairs Medical Center (VAMC) during COVID-19 Pre-pandemic and Pandemic periods: A 4-year Analysis and Implications for Antimicrobial Stewardship

**DOI:** 10.1093/ofid/ofad500.1852

**Published:** 2023-11-27

**Authors:** Sajjad Ali, Ryan Kuhn, Sumaiya F Khaja, Suganthini Krishnan Natesan

**Affiliations:** Wayne State University, Detroit, Michigan; John D Dingell VA Medical Center, Detroit, Michigan; Wayne State University School of Medicine, Rochester Hills, Michigan; John D Dingell VAMC/Wayne State University, Detroit, Michigan

## Abstract

**Background:**

During the coronavirus disease 2019 (COVID-19) pandemic, antimicrobial use increased tremendously. Evidence suggests that the pandemic has resulted in an increased rate of AMR due to inadvertent antibiotic use. This likely reflects difficulties in distinguishing COVID-19 from community-acquired pneumonia when patients first arrive at a hospital. Strengthening antimicrobial stewardship (AMS) efforts by formulating policies for the use of antibiotics is crucial to prevent emergence of AMR.

**Methods:**

A retrospective analysis to assess changes in antibiotic use during the pandemic and pre-pandemic patterns was done.

**Results:**

: In the **inpatient setting**, there was an overall decrease in antibiotic use (all units that follow are in DOT/1000 BDOC) from an average of 380 to 300, vancomycin from 90 to 62, cefazolin from 30 to 17, Linezolid from 5 to 2, Zosyn from 57 to 30 from pre-pandemic period (Jan 2019) to pandemic period (Dec 2022). Antimicrobials that showed an increase during the pandemic included ceftriaxone from 70 to 82, Cefepime from 5 to 8, Levofloxacin from 2 to 5, and fluconazole from 4 to 10 compared to pre-pandemic use. In the **outpatient settings** (units are in fills/1000 unique patients), levofloxacin use doubled from 3 to 6, cefuroxime use increased from 0.5 to 2 and Azithromycin use dropped from 80 to < 20, with concomitant increase in doxycycline use from 45 to 60. Outpatient antibiotic prescriptions were high for levofloxacin, doxycycline and cefuroxime and increases corresponded to peaks in cases of COVID-19.

**Conclusion:**

Our analysis demonstrates that COVID-19 had shifted the antibiotic consumption curve towards treatment of pneumonia and invasive fungal infections (in seriously ill patients) which has key implications for AMS. Implementation of stringent AMS and mandatory Infectious disease consultation for all COVID 19 infections helped keep our antibiotic consumption well below the national average and curtail AMR.

**Disclosures:**

**All Authors**: No reported disclosures

